# Clinicopathological analysis of T2-FLAIR mismatch sign in lower-grade gliomas

**DOI:** 10.1038/s41598-020-67244-7

**Published:** 2020-06-22

**Authors:** Shoichi Deguchi, Takuma Oishi, Koichi Mitsuya, Yuko Kakuda, Masahiro Endo, Takashi Sugino, Nakamasa Hayashi

**Affiliations:** 1 Division of Neurosurgery, Shizuoka Cancer Center, Shizuoka, Japan; 2 Division of Pathology, Shizuoka Cancer Center, Shizuoka, Japan; 3 Division of Diagnostic Radiology, Shizuoka Cancer Center, Shizuoka, Japan

**Keywords:** Cancer imaging, CNS cancer

## Abstract

T2-FLAIR mismatch sign is known as a highly specific imaging marker of IDH-mutant astrocytomas. This study was intended to clarify what the T2-FLAIR mismatch sign represents by pathological analysis of lower-grade gliomas rediagnosed in accordance with the WHO 2016 classification. We retrospectively analyzed the records of 64 patients diagnosed with WHO grade II and III diffuse gliomas between June 2009 and November 2018. T2-FLAIR mismatch sign was found in 10 (45%) out of 22 patients with IDH-mutant astrocytoma, 1 (5%) out of 20 with oligodendroglioma, and 1 (5%) out of 22 with IDH-wild-type astrocytoma. T2-FLAIR mismatch sign as a marker of IDH-mutant astrocytomas showed positive predictive value of 83%. Among 22 patients with IDH-mutant astrocytomas, microcystic change was found in eight, of which seven showed T2-FLAIR mismatch sign. Microcystic change was significantly associated with T2-FLAIR mismatch sign (P < 0.01). From multi-sampling in a patient, abundant microcysts were observed upon HE staining of specimens from the T2-FLAIR mismatched region, while microcysts were hardly observed from the T2-FLAIR matched one. All three protoplasmic astrocytomas among our IDH-mutant astrocytomas presented T2-FLAIR mismatch sign. In conclusion, T2-FLAIR mismatch sign may reflect microcyst formation in IDH-mutant astrocytomas and be common in IDH-mutant protoplasmic astrocytoma.

## Introduction

Diffuse lower-grade gliomas are diagnosed by histopathological and molecular features, such as isocitrate dehydrogenase (*IDH*) 1 and 2 gene mutation and codeletion of chromosomes 1p and 19q (1p19q codeletion) in the World Health Organization (WHO) 2016 classification^1,2^. Oligodendrogliomas harbor both IDH mutation and 1p19q codeletion. IDH-mutant astrocytomas harbor IDH mutation but not 1p19q codeletion, and IDH-wild-type astrocytomas harbor neither of them. Oligodendroglioma show improved sensitivity to chemotherapy^3,4^ and a more favorable clinical outcome than gliomas without 1p19q codeletion^5,6^. IDH-wild-type astrocytomas show a worse clinical outcome than gliomas with IDH mutation^5,6^. IDH-mutant astrocytomas show an intermediate clinical outcome^5,6^. Therefore, preoperative identification of these molecular subtypes is useful for surgical planning, setting the treatment strategy, and prognostication.

T2-FLAIR mismatch sign, which is defined as the presence of complete/near-complete hyperintense signals on a T2-weighted image and a relatively hypointense signal on FLAIR except for a hyperintense peripheral rim, has been suggested^7^ and validated as a highly specific imaging marker of IDH-mutant astrocytomas, with specificity as high as 100%^8^. However, little is known about the pathological finding associated with T2-FLAIR mismatch sign.

The purpose of this study is to clarify what the T2-FLAIR mismatch sign represents by pathological analysis of lower-grade gliomas rediagnosed in accordance with the WHO 2016 classification.

## Materials and methods

### Patient selection and clinical data

The records of 91 patients who were consecutively diagnosed with WHO grade II and III diffuse gliomas at Shizuoka Cancer Center between June 2009 and November 2018 were collected. Among them, we retrospectively analyzed the clinical, radiological, and pathological data of 64 patients in this study, using the following inclusion criteria: availability of (1) preoperative MR imaging data with FLAIR and T2-weighted image and (2) molecular information of IDH1 mutation and 1p19q codeletion status. Sixty-four patients were rediagnosed in accordance with the WHO 2016 classification.

Clinical information was obtained retrospectively from the patients’ electronic medical records. The clinical data included age at diagnosis, sex, histological type, side/location of tumor, extent of tumor resection, date of the surgery, date when the first clinical or radiological evidence of tumor progression was found, and date of death or last follow-up visit. The institutional review board of Shizuoka Cancer Center approved this retrospective study, and the requirement for informed consent was waived. All methods were performed in accordance with the relevant guidelines and regulations.

### Pathological diagnoses and molecular classification

Pathological diagnoses were performed by two experienced neuropathologists in a routine, histological manner using hematoxylin–eosin-stained sections in accordance with the WHO criteria. IDH1 Arg132His mutations were examined by immunohistochemistry (IHC) with a mutation-specific antibody (internal clone H14; Dianova, Hamburg, Germany).

The 1p19q codeletion status was determined by fluorescence *in situ* hybridization with specific probe for 1p36 and 19q13 foci. The 1p19q codeletion was defined as the deletion of >50% of the nuclei examined for both 1p and 19q^9^.

### Imaging analysis

**Figure 1:**
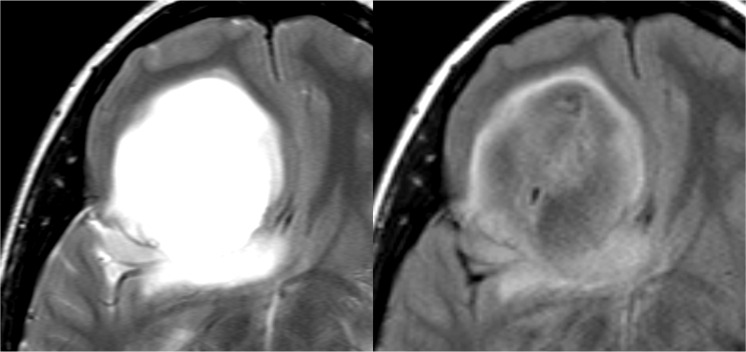
An example of T2-FLAIR mismatch sign. A patient with IDH-mutant astrocytoma in the right frontal lobe. Left shows T2-weighted image and right shows FLAIR image.

MR scans were performed at 1.5 T or 3.0 T. T2-FLAIR mismatch sign was independently evaluated by two experienced reviewers: board-certified neurosurgeons with 13 (S.D.) and 26 (K.M.) years’ experience. The two reviewers were blinded to each other results. In the case of discordance, the two reviewers discussed the findings until agreement was reached. Further, an experienced radiologist (M.E.) confirmed the review by the two neurosurgeons. T2-FLAIR mismatch sign was defined as in a previous report^7^: the presence of complete/near-complete hyperintense signals on T2-weighted image and relatively hypointense signal on FLAIR except for a hyperintense peripheral rim (Fig. 1).

### Multi-sampling technique

**Figure 2:**
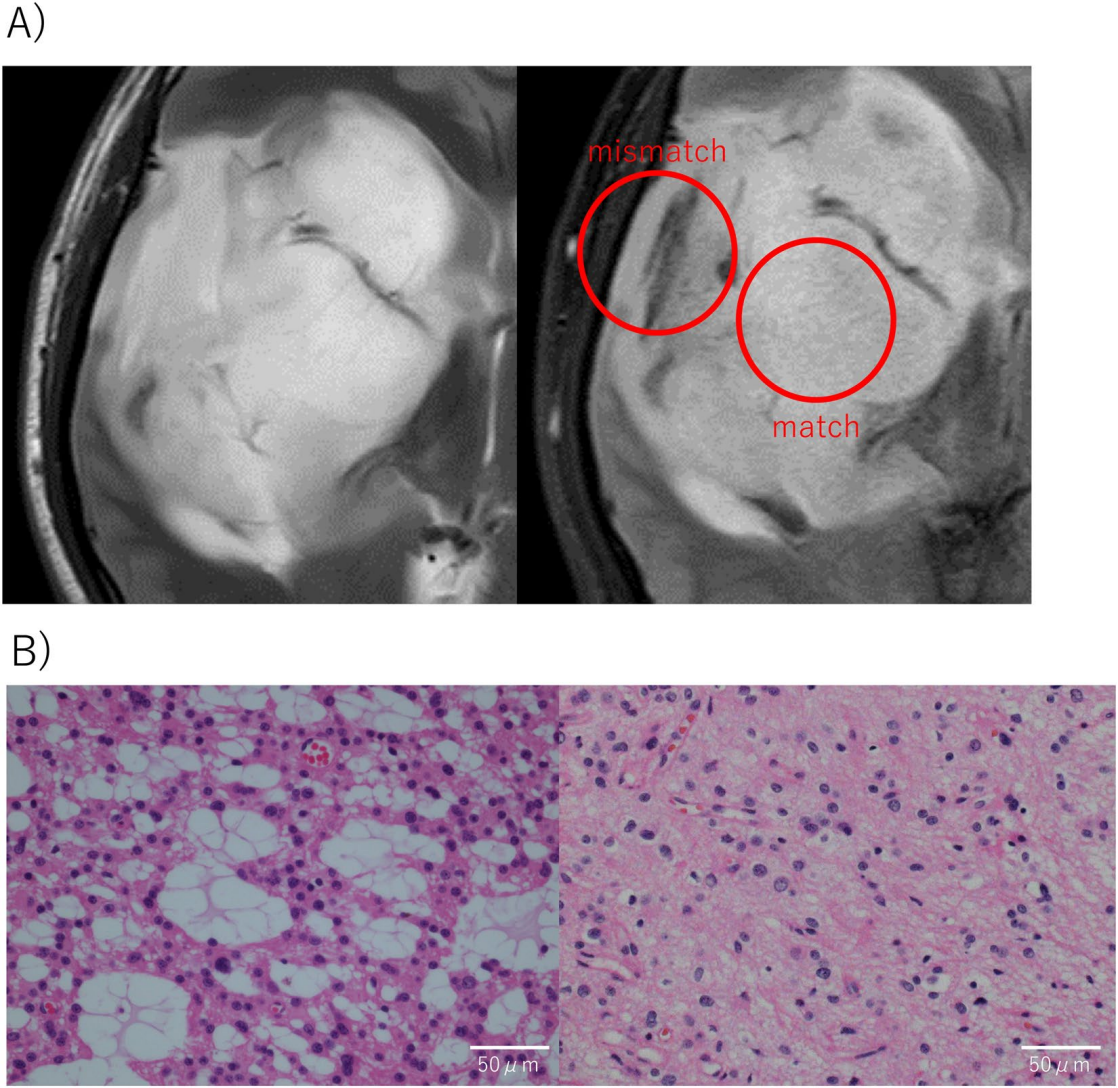
Multi-sampling in a patient with IDH-mutant astrocytoma. (**A**) MR imaging. Left shows T2-weighted image and right shows FLAIR image. Red circles show T2-FLAIR matched and mismatched regions. (**B**) Hematoxylin–eosin (HE)-stained sections. Left shows HE staining of specimens from the T2-FLAIR mismatched region, where multiple microcysts were observed. Right shows HE staining of specimens from the T2-FLAIR matched region, where microcysts were hardly observed.

We performed multi-sampling in a patient with IDH-mutant astrocytoma consisting of matched and mismatched regions in T2-weighed image and FLAIR (Fig. 2A). MRI revealed that the tumor contained T2-FLAIR mismatch sign near the surface but not in the deep part. The specimens were separately removed from both regions, and were pathologically analyzed.

### Statistical analysis

Statistical analyses were performed using EZR statistical software^10^. Progression-free survival (PFS) was defined as the time from surgery to the first clinical or radiological evidence of tumor progression. Overall survival (OS) was defined as the time from surgery to death or the last follow-up. A univariate analysis was conducted with Fisher’s exact test. PFS and OS were calculated using the Kaplan–Meier method and compared using log-rank test. P < 0.05 was considered statistically significant.

## Results

### Patients’ characteristics

We analyzed 64 patients with lower-grade gliomas who were rediagnosed in accordance with the WHO 2016 classification (Table 1). They included 20 cases of oligodendroglioma (IDH1 mutation and 1p19q codeletion), 22 cases of IDH-mutant astrocytoma (IDH1 mutation and 1p19q non-codeletion), and 22 cases of IDH-wild-type astrocytoma (IDH1 wild type and 1p19q non-codeletion). Median ages at diagnosis in these groups were 46 (29–74), 39 (20–65), and 57 (22–80) years old, respectively. Moreover, 13 (65%), 17 (77%), and 8 (36%) patients were respectively diagnosed with WHO grade II glioma.

**Table 1:** Characteristics of patients with lower grade gliomas.

	N	Male/Female	Median age (range)	WHO Grade II/III	T2-FLAIR mismatch sign Present/Absent
IDH1 mut/1p19q non-codel	22	14/8	39 (20–65)	17/5	10/12
IDH1 mut/1p19q codel	20	9/11	46 (29–74)	13/7	1/19
IDH1 wild type	22	15/7	57 (22–80)	8/14	1/21

Abbreviations: IDH1: isocitrate dehydrogenase 1, N: number, WHO: World Health organization.

### T2-FLAIR mismatch sign as an imaging marker

The inter-rater agreement for the T2-FLAIR mismatch sign was good (κ = 0.73). Six discordant cases (9.4%) were reassessed independently by both reviewers. This MRI assessment was confirmed by the third reviewer. T2-FLAIR mismatch sign was found in 10 (45%) out of 22 patients with IDH-mutant astrocytomas, whereas it was found in 1 (5%) out of 20 patients with oligodendrogliomas and 1 (5%) out of 22 patients with IDH-wild-type astrocytomas (Table 1). T2-FLAIR mismatch sign as a marker of IDH-mutant astrocytomas showed positive predictive value of 83% and negative predictive value of 77%. Sensitivity and specificity were 46% and 95%, respectively (Table 2).

**Table 2:** T2-FLAIR mismatch sign as an imaging marker.

Sensivity	0.46 (95%CI: 0.24–0.68)
Specificity	0.95 (0.84–0.99)
PPV	0.83 (0.52–0.98)
NPV	0.77 (0.63–0.88)

Abbreviations: PPV: positive predictive value, NPV: negative predictive value, 95%CI: 95% confidence interval.

### Clinical features of T2-FLAIR mismatch sign in IDH-mutant astrocytomas

**Figure 3:**
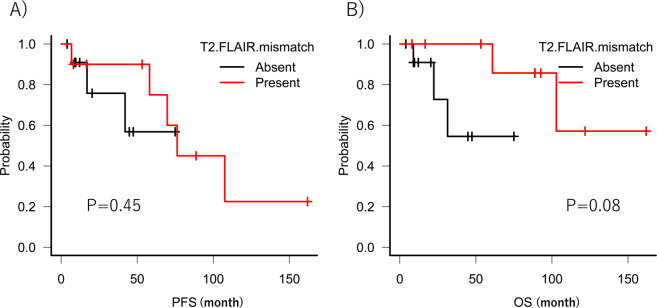
Kaplan–Meier curves of progression-free survival (PFS) and overall survival (OS). (**A**) Kaplan–Meier curves of PFS in IDH-mutant astrocytomas comparing the presence of T2-FLAIR mismatch sign (red line) with its absence (black line). (**B**) Kaplan–Meier curves of OS in IDH-mutant astrocytomas comparing the presence of T2-FLAIR mismatch sign (red line) with its absence (black line).

Univariate analysis was performed to determine whether clinical factors affected the T2-FLAIR mismatch sign in IDH-mutant astrocytomas (Table 3). Age at diagnosis, sex, and side/location of tumor were not significantly associated to T2-FLAIR mismatch sign. In addition, univariate analysis of PFS and OS was performed. The presence or absence of T2-FLAIR mismatch sign did not significantly affect PFS and OS (P > 0.05) (Fig. 3A,B).

**Table 3:** Clinicopathological features of T2-FLAIR mismatch sign in IDH-mutant astrocytomas.

	T2-FLAIR mismatch sign		P value
	Present (10)	Absent (12)	
Sex: Men	5	9	0.34
Age < Median: 36 y.o	6	5	0.67
WHO Grade II	7	10	0.62
Tumor location (Right)	7	6	0.56
Tumor location (Frontal lobe)	9	10	1.00
Pathology (Microcystic change)	7	1	<0.01

Abbreviations: IDH1: isocitrate dehydrogenase 1, y.o: years old, WHO: World Health Organization.

### Pathological findings of T2-FLAIR mismatch sign in IDH-mutant astrocytomas

Pathological analysis of 22 patients with IDH-mutant astrocytomas was performed. Microcystic change was found in eight patients, of which seven showed T2-FLAIR mismatch sign. Microcystic change was significantly associated with T2-FLAIR mismatch sign (P < 0.01) (Table 3). Three of the ten patients with IDH-mutant astrocytomas who exhibited T2-FLAIR mismatch sign did not show microcystic change pathologically. In two of these three patients, post-operative MRI showed that the specimens of the tumor were not removed from the region with the T2-FLAIR mismatch sign. Regarding the histopathological classification of astrocytoma, the ten patients who exhibited T2-FLAIR mismatch sign included five with fibrillary astrocytoma, three with protoplasmic astrocytoma, and two with gemistocytic astrocytoma. Although 22 patients with IDH-mutant astrocytomas included three protoplasmic astrocytomas, all of them presented T2-FLAIR mismatch sign.

### Multi-sampling in a patient with IDH-mutant astrocytoma

We performed multi-sampling in a patient with IDH-mutant astrocytoma consisting of matched and mismatched regions in T2-weighed image and FLAIR (Fig. 2A). Multiple microcysts were observed in HE staining of specimens from the T2-FLAIR mismatched region, while microcysts were hardly observed in the specimens from the T2-FLAIR matched region (Fig. 2B).

## Discussion

The development of radio-genomic signatures in lower-grade gliomas has been progressing in recent years^11^. It has been reported that ADC values associate with IDH mutation status^9,12^. IDH-wild-type astrocytomas were shown to have significantly higher cerebral blood volume than IDH-mutant astrocytomas^9,12^. 2-Hydroxyglutarate MR spectroscopy demonstrated high specificity for prediction of IDH-mutant glioma^13^. The 1p19q codeletion has also been found to be associated with T2-signal heterogeneity, subcortical involvement, frontal lobe location, and calcification^14,15^. T2-FLAIR mismatch sign, one of the radio-genomic signatures, is reportedly specific for IDH-mutant astrocytomas, with specificity as high as 100%^7,8,16^. Indeed, in our cohort, T2-FLAIR mismatch sign represented a highly specific imaging marker for IDH-mutant astrocytomas, with specificity of 95%, but our cohort included two false-positive cases. Lee *et al*.^9^ and Juratli *et al*.^11^ also presented false-positive cases of T2-FLAIR mismatch sign in adult diffuse lower-grade gliomas. In addition, Johnson *et al*. introduced four false-positive cases in a pediatric population, which included pilomyxoid astrocytoma, lower-grade astrocytoma harboring MYB rearrangement, H3K27M-mutant midline glioma, and non-neoplastic region^17^. It may be challenging to use T2-FLAIR mismatch sign alone instead of biopsy for diagnosis of IDH-mutant astrocytoma. We consider that the combination of simple radio-genomic signatures in lower-grade gliomas may be more accurate and helpful for preoperative diagnostic imaging than only T2-FLAIR mismatch sign.

The reason why the phenotypes in FLAIR image differ in the same IDH-mutant astrocytomas remains unclear. In our cohort, about half of IDH-mutant astrocytomas did not show T2-FLAIR mismatch sign, which is consistent with previous reports^7,8^. We could not identify clinical factors (sex, age, WHO grade, tumor location) associated with the T2-FLAIR mismatch sign in this study. In addition, there was no significant difference in PFS or OS depending on the presence or absence of T2-FLAIR mismatch sign. These findings are consistent with a previous report^8^. A detailed pathological and genetic analysis about the difference of FLAIR image in IDH mutant astrocytomas is required.

T2-FLAIR mismatch sign was significantly associated with the pathological finding of microcystic change. In terms of genetics, Patel *et al*. assumed that T2-FLAIR mismatch sign may be associated with increased levels of proteins involved in the mammalian target of rapamycin pathway. However, their data were only preliminary and not validated^7^. In terms of pathology, Tay *et al*. reported that all eight consecutive cases of histologically diagnosed protoplasmic astrocytoma demonstrated T2-FLAIR signal suppression^18^. Indeed, all of three protoplasmic astrocytomas among our IDH-mutant astrocytomas presented T2-FLAIR mismatch sign. These findings suggest that T2-FLAIR mismatch sign reflects microcystic change, since microcystic change is a histological hallmark of protoplasmic astrocytoma. Patel *et al*. performed histopathological analysis of 30 patients with IDH-mutant astrocytomas. They concluded that there was a non-significant trend for the presence of abundant microcysts in the T2-FLAIR mismatch-positive cases compared with the T2-FLAIR mismatch-negative ones (P = 0.128)^7^. In the present study, in order to evaluate association with microcystic changes in pathological findings and T2-FLAIR mismatch in MR imaging, we performed multi-sampling in a patient with IDH-mutant astrocytoma. We observed microcyst formation only from the area of T2-FLAIR mismatch sign.

Our study has several limitations. First, the retrospective nature of our analysis is associated with patient selection bias. Specifically, we excluded the patients without appropriate MR imaging with FLAIR and T2-weighted image or molecular information of IDH1 mutation and 1p19q codeletion status in accordance with the WHO 2016 classification. Second, there was variability in the MRI platforms and imaging parameters between some patients across the studies, which may affect the MRI assessment. Last, our study included a relatively small number of cases due to the rarity of diffuse lower-grade gliomas. We need to histologically validate the relationship between T2-FLAIR mismatch sign and microcyst formation in more cases.

## Conclusions

We demonstrated that T2-FLAIR mismatch sign may reflect microcyst formation in IDH-mutant astrocytomas and be common in IDH-mutant protoplasmic astrocytoma. We need to validate our findings with more cases.
